# The synergistic evolution of supply-demand composite system for airport green development: A case study in Guangzhou Baiyun International Airport, China

**DOI:** 10.1371/journal.pone.0302303

**Published:** 2024-04-30

**Authors:** Lili Wan, Yangyang Lv, Zhan Wang, Yong Tian

**Affiliations:** College of Civil Aviation, Nanjing University of Aeronautics and Astronautics, Nanjing, China; Al Mansour University College-Baghdad-Iraq, IRAQ

## Abstract

Given the pressing requirements for sustainable development in civil aviation, conducting a synergistic evolution analysis of the supply and demand aspects in the airport green development holds great significance. This analysis helps achieve sustainable airport development and facilitates the green transformation of civil aviation development. Taking a collaborative learning approach and utilizing historical data from Guangzhou Baiyun International Airport spanning 2008 to 2019, the supply-demand composite system for airport green development was deconstructed into two subsystems—demand and supply—and relevant evaluation index systems were established in this paper. A screening and optimization model of supply and demand synergy indicators for airport green development was constructed, and it was solved using a simulated annealing genetic algorithm. The Haken model was constructed to analyze the synergistic evolutionary relationship of the composite system of supply and demand for green airport development in two stages. The results indicate a shift in the order parameter of the co-evolution of the supply-demand composite system at Guangzhou Baiyun International Airport, moving from the demand subsystem in the first stage (2008–2015) to the supply subsystem in the second stage (2016–2019). The co-evolution of the airport supply-demand composite system has entered a new stage, but has not reached a high level of synergy. The study not only contributes theoretically by explaining the interaction mechanism between supply and demand for airport green development, but also offers targeted suggestions for achieving high-quality synergistic evolution of supply and demand for airport green development.

## Introduction

The co-evolution of the demand and supply sides is crucial for the sustained and stable development of the supply-demand composite system. However, diverse and dynamic transportation demands exacerbate inherent limitations and time delays in transportation supply, leading to an imbalance in air transport’s supply and demand. This imbalance results in critical issues such as airspace congestion, increased workload for air traffic controllers, and severe environmental pollution. These challenges significantly impede the sustainable development of the air transport industry. Consequently, the Civil Aviation Administration of China (CAAC) has devoted two decades to the advancement of the air transportation industry. Addressing these challenges, the CAAC unveiled the "14th Five-Year Plan for Green Development of Civil Aviation" in 2021. This plan aims to improve the green civil aviation supply system to facilitate the demands of eco-friendly civil aviation development, with the goal of realizing an eco-friendly, low-carbon, and sustainable evolution of civil aviation.

Airports are critical infrastructures for the air transportation industry, promoting trade exchanges and economic prosperity. However, their operational activities also lead to the generation of air and noise pollution, posing significant environmental threats. This challenge arises from the misalignment between the airport supply strategy, primarily oriented toward economic development, and the demand for green development based on resource conservation and environmental protection. Hence, conducting an in-depth analysis of the supply and demand dynamics of airports during the green development process, elucidating the interaction mechanism of the supply-demand composite system, holds immense research significance. This is crucial for establishing a high-level dynamic equilibrium between demand-driven supply and supply-adapted demand, ultimately contributing to the sustainable development of airports.

Airports, as critical nodes in the air transportation network, are confronting escalating challenges such as airspace congestion and flight delays. These issues arise from the mismatch between the continuous growth of air traffic and the constrained operational capacity of airports. Consequently, airports have emerged as significant bottlenecks in air traffic operations, accentuating the contradictions within the airport supply and demand relationship [1]. To address these challenges, the majority of scholars are presently concentrating on research related to the capacity and flow coordination of airport operations. This primarily encompasses adjustments to flight schedules [2], optimization of airspace structure [3, 4], and coordination in air traffic flow management [5]. Following the integration of the concept of sustainable development into airport development in the "13th Five-Year Plan" for civil aviation, some scholars have shifted their focus to the perspective of green development to analyze the supply and demand relationship of airports. However, current research predominantly emphasizes the unilateral study of supply and demand, examining the driving factors of airport green development from the demand perspective [6] or assessing and optimizing the environmental carrying capacity of airport green development from the supply standpoint [7, 8]. There is a scarcity of studies exploring the coordinated relationship between the supply and demand sides of airport green development.

The supply-demand composite system for airport green development consists of diverse supply and demand elements. Green airport development of is fundamentally a process in which this elements achieve coordinated development through interaction. In this process, research on the supply and demand sides is constrained by the development levels of each side, posing challenges in analyzing the supply-demand evolution relationship of airports in the process of green development from the comprehensive standpoint of the supply-demand composite system [9]. To establish a green and low-carbon airport development system and promote the comprehensive green transformation of civil aviation development, green development should be integrated into the airport supply-demand composite system. Exploring the feasible path of airport green development from the perspective of the synergistic evolution between supply and demand sides is crucial.

In the study of the coordinated evolution of green airport development supply and demand, effectively assessing the current situation of green airport development supply and demand is a prerequisite for analyzing the coordinated evolution of supply and demand on both sides [10]. Thus, conducting a thorough analysis of the supply and demand dimensions of airport green development is crucial. This analysis should commence by precisely defining the significance of supply and demand in airport green development. Only through accurate evaluation of the levels of supply and demand can we proceed to analyze the synergistic evolution process in the context of airport green development. This, facilitates the implementation of targeted measures to establish a high-level coordinated development pattern for airport green development and achieve the transformation into a green airport. Thus, given the current state and its limitations of existing research, this paper adopts a synergistic theory-based approach to investigate the synergistic evolution of the supply-demand composite system for airport green development. In comparison with existing studies, the primary contribution of this article is:

1. Drawing on the distinctions in elements and structures between land transportation and air transportation, and in alignment with the concept of sustainable development in civil aviation, we analogize the supply and demand connotations of airport green development with the definition of urban transportation supply and demand.
2. Through deconstructing the complex system of airport green development supply and demand, this study, from the standpoint of system theory and synergy, establishes the self-organized characteristics of simultaneous evolution in airport green development supply and demand. Additionally, it constructs respective index systems for airport green development supply and demand. Furthermore, the entropy weight linear weighting method is applied to measure the supply and demand levels of airport green development.
3. This study constructs a synergistic evolution model of the supply-demand composite system for airport green development. It analyzes the interaction and coordinated development process between supply and demand, which is of great practical significance for achieving high-quality supply and demand synergy for airport green development.

The main research content of this paper is structured as follows: The second part introduces the relevant literature, reviews previous research findings and the current research status, thereby establishing the groundwork for the advancement of this study. The third part defines the connotation of green airport development supply and demand, proves the self-organization characteristics of the supply-demand composite system, establishes the index system of supply and demand for airport green development, and further constructs the co-evolution model of supply and demand for airport green development. The fourth part analyzes the synergistic evolution of the supply and demand sides of the green development of Guangzhou Baiyun International Airport (CAN) in two stages. The fifth part draws conclusions and writes policy recommendations.

## Literature review

The synchronized advancement of supply and demand is a pivotal issue in economic and social progress. Originating in economic theory to analyze the interaction between supply and demand for goods [11], this concept has expanded its scope to encompass the transportation sector. Scholarly interest has been steadily growing in research aimed at aligning transportation flow demand with transportation structure service capacity, particularly when viewed through the lens of supply and demand matching [12].

The study of transport supply and demand begins with the field of ground transportation. In the definition of the transportation supply and demand concept, Duan outlines urban and rural transportation demand as the spatial movement of passengers and goods facilitated by road network transport. Correspondingly, urban and rural transportation supply is characterized as the road network transport system that sustains the spatial movement within urban and rural areas [13]. Zhang established dynamic passenger demand model and dynamic service supply model in order to compare and analyze the supply and demand levels of urban rail transit systems from the perspectives of demand and supply [14]. In their exploration of the interplay between traffic supply and demand, Li and colleagues devised a "double-chain" rail transit industry chain system model. They employed a two-dimensional composite coordination model to assess the coordinated evolution level of the rail transit industry chain system in Tangshan City [15].

Subsequently, scholars expanded their examination of transportation supply and demand from ground transportation to air traffic, delving into the processes of aligning capacity and flow in airport operations. Zhao introduced the concept of adaptability in airport construction scale economy and conducted an analysis of the conditions for achieving the optimal state of airport construction scale that aligns with socio-economic factors [16]. Utilizing the operational data from Chengdu Shuangliu Airport, He employs the capacity envelopment method to estimate both the actual and ultimate capacities of the airport [17]. Following the integration of the sustainable development concept, scholars have initiated the examination of airport supply and demand relationships through the lens of green development. Liang established a robust green airport evaluation index system using the DPSIR model. This involved defining the concept of green airports and summarizing the characteristic elements of green airport development [18]. Subsequently, an analysis of the driving factors behind the requirements for airport green development was conducted. Approaching from the standpoint of environmental pollution and considering the LTO (Landing and Takeoff) cycle emissions of single-engine aircraft, Wang formulated a macroscopic estimation for airport environmental carrying capacity [19]. Wan introduced the definition of airport environmental carrying capacity, formulated the evaluation index system for airport environmental carrying capacity, and performed an assessment and prediction of the environmental carrying capacity of Guangzhou Baiyun International Airport [20].

In conclusion, research on supply and demand relationships in the transportation sector has yielded significant outcomes, but there are still some gaps in the research on airport green development supply and demand. Firstly, existing research lacks analysis of the dynamics of supply and demand in the field of air traffic, making it challenging to define the concept of airport green development supply and demand. Secondly, the majority of studies on airport supply-demand relationships have emphasized capacity matching. However, research on airport supply-demand relationships from the perspective of green development primarily involves one-sided analysis of supply and demand. There is limited research on the synergistic evolution of airport supply and demand in the process of green development from the overall perspective of the supply-demand composite system.

Synergistic evolutionary analysis in composite systems exploration can investigate the interactive relationships and coordinated developmental processes among subsystems [21]. The concept of coevolution emerged in the 1970s with Herman Haken’s notion of synergetics, highlighting complex systems’ ability to internally coordinate and cooperate through self-organization, leading to systematic and ordered development [9]. In this process, the Haken model can identify the dominant co-evolutionary parameters of the composite system using the approximate adiabatic assumption mechanism [22]. This enables the analysis of the interaction and coordinated development processes between subsystems. Zhong investigates the synergistic evolution of economic growth and energy consumption in the Beijing-Tianjin-Hebei region by analyzing the stage changes of the control variables in the Haken model [23]. Du identified the sequential covariates of the synergistic development between the logistics industry and the manufacturing industry using the two-stage Haken model. They also summarized the patterns observed in the synergistic development of the logistics and manufacturing industries in the Yangtze River Economic Belt [24]. Chen and colleagues developed an evaluation index system for the water-energy-food system of the Yangtze River Economic Belt using the PSR model. They employed the Haken model to identify the driving factors influencing the coordinated evolution of the dominant system. This was utilized as a key variable in simulating scenarios for certain provinces in the Yangtze River Economic Belt in 2025 [25].

Therefore, addressing the deficiencies and shortcomings of existing research, this article adopts the concept of urban transportation supply and demand to analogize the connotation of airport green development supply and demand. It constructs the evaluation index system for airport green development supply and demand respectively. From the perspective of synergy research, this paper chooses airport green development supply and demand synergy indicators based on the approximation adiabatic conditions of the Haken model. The synergistic evolutionary process of the airport green development supply and demand composite system is studied in stages, aiming to offer suggestions for promoting the high-quality synergy evolution of airport green development supply and demand.

## Research and data methodology

### 3.1. Connotation and deconstruction

As airports streamline their organizational roles and functions, the concept of green airport development has evolved into a sophisticated and integrated and complex. Achieving a profound understanding of its essence and conducting a detailed analysis of the supply and demand relationship that facilitates this eco-friendly transformation, it becomes essential to deconstruct the supply-demand composite system for airport green development (abbreviation for the SDCS) [13].

From the perspective of supply and demand, the demand for airport green development encompasses the goals and driving factors necessary to achieve eco-friendly transformation in airports. Conversely, the supply for airport green development comprises the supporting and guaranteeing elements required to realize such eco-friendly goals [10]. The relationship between the supply and demand sides of airport green development is dialectical and unified. On one hand, the supply and demand sides are closely intertwined and mutually reinforcing. The green development supply determines the objectives, quality, and approach of green development demand. In turn, green development demand establishes various requirements for green development supply, leading to the enhancement of supply levels and structural adjustments. On the other hand, the supply and demand sides of airport green development maintain a certain degree of independence. The scale and approach of green development supply are closely influenced by regional socio-economic legacies, green development ideologies, and the level of environmental protection technology. Simultaneously, the green development demand is shaped collectively by national development strategies, green development philosophies, and development policies.

From the standpoint of systems theory, the green development of airports is a complex process grounded in the coordination of the demand sub-system(abbreviation for the DSS) and supply sub-system(abbreviation for the SSS) [26]. The green development process essentially represents an evolutionary journey of the SDCS, transitioning from disorder to order and from a low level to a high level through the mechanism of self-organization [27]. This evolutionary mechanism exhibits self-organized synergistic evolutionary properties.

The SDCS exhibits openness, characterized by resource and information exchanges between its elements and the external socio-economic environment. Moreover, the SDCS displays nonlinearity, containing multiple subsystems with diverse characteristics, and complex interrelationships among its elements. Changes in the system due to alterations in a state variable do not follow a proportional linear relationship but rather emerge through a nonlinear mechanism. Under the joint action of openness and nonlinearity, there are differences in the spatial flow of the elements, forming a composite system far from equilibrium, which is manifested as the imbalance of the supply and demand composite system. Under this state of imbalance, the system’s elements possess different information potentials, which externalize as a gradient supply and demand difference within the system. Consequently, the system experiences various stochastic ups and downs along the gradient direction. Therefore, the SDCS is an open, nonlinear system with fluctuations and far from the equilibrium state [28].

**Fig 1:**
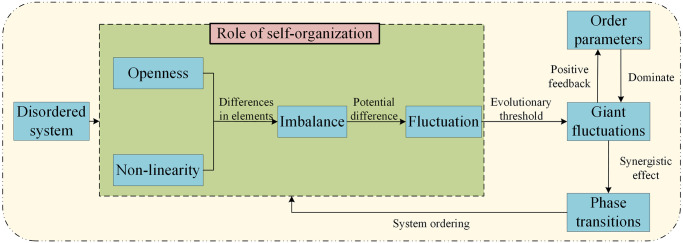
Synergistic evolutionary processes in composite systems.

During the synergistic evolution of the SDCS, fluctuations dominated by fast relaxation variables undergo large damping motions and rapidly decay since they are not effectively responded to by other subsystems. As the evolution approaches a threshold, stronger nonlinear effects lead some fluctuations to spread across the entire system, resulting in giant fluctuations due to the responses of subsystems. These fluctuations are primarily influenced by order parameter and generate phase transitions through synergistic effects. This process drives the system to form a new macroscopic structure, thereby achieving orders [25]. As shown in Fig 1:

### 3.2. Supply and demand index system for airport green development

Based on the connotation of airport green development supply and demand, according to the principles of hierarchy, scientificity, operability, and combination of qualitative description and quantitative calculation in the construction of evaluation indexes, we construct the evaluation index system of airport green development supply and demand in this paper.

#### 3.2.1. Demand index system for airport green development

As per the "Guidelines for Green Airport Planning" issued by the CAAC in 2018 [29] and the " Four Characteristics Airport Development Guidelines " (abbreviation for the Guidelines) issued in 2020 [30], the construction of green airports should be guided by the principles of resource conservation, environmental protection, and providing efficient, convenient, and humanized services. The demand for airport green development is therefore categorized into three main aspects: resource conservation, environmental protection and efficient operation. The indicators used to assess these aspects are mainly drawn from the " Code for the Green Performance Assessment of Four Characteristics Airport" [31] and the "14th Five-Year Plan for Specialized Green Development of Civil Aviation"(abbreviation for the Plan) [32].

Based on the Guidelines and the Plan, this paper constructs resource conservation layer indicators that focus on three aspects: land intensification, water conservation, and energy conservation. The environmental protection layer indicators are developed with a focus on carbon emissions and pollutant emissions. And the efficient operation layer indicators are selected from the production system and operational efficiency of airports.

In summary, the three-dimensional "Resource-Environment-Operation" (abbreviation for the REO) airport green development demand index system is shown in Table 1:

**Table 1:** Demand index system for green development of airports.

System	Subsystem	Sequence	Indicators	Unit	Attributes
Demand subsystem	R	*x*_1_	Annual passenger throughput per unit land area	10000 persons/km^2^	Positive
		*x*_2_	Annual takeoff and landing sorties per unit land area	10000 sorties/km^2^	Positive
		*x*_3_	Annual average unit passenger comprehensive water consumption	Liter/person	Negative
		*x*_4_	Annual average unit passenger comprehensive energy consumption	Kilogram standard coal/person	Negative
	E	*x*_5_	Annual CO_2_ emissions per unit passenger throughput	g/person	Negative
		*x*_6_	Annual CO emissions	g	Negative
		*x*_7_	Annual HC emissions	g	Negative
		*x*_8_	Annual NOx emissions	g	Negative
		*x*_9_	Annual PM emissions	g	Negative
		*x*_10_	Noise pollution	dB	Negative
	O	*x*_11_	Annual passenger throughput	person	Positive
		*x*_12_	Annual cargo throughput	ton	Positive
		*x*_13_	Annual aircraft movements	sorties	Positive
		*x*_14_	Airport clearance rate	%	Positive
		*x*_15_	Average airport taxi time	minute	Negative

#### 3.2.2. Supply index system for airport green development

The PSR model, also known as the "Pressure-State-Response" model, is a widely used evaluation model in the field of environmental quality assessment [33]. This paper applies the PSR model to the context of airports green development supply and establishes a dynamic analysis framework that examines the pressure, state, and response for the airport green development supply.

**Fig 2:**
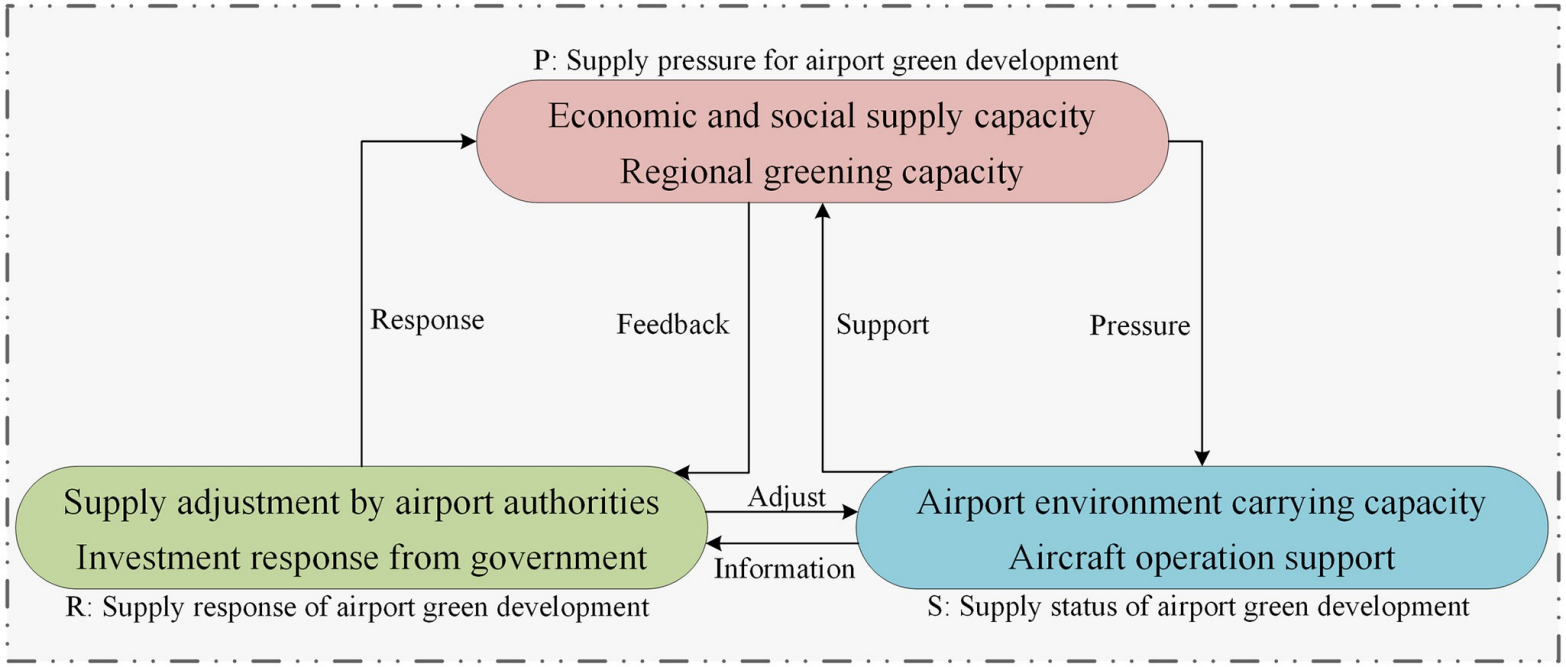
PSR modeling for green development provisioning of airports.

In the process of aligning supply and demand for green airport development, the airport green development supply takes into account both the macro-environment of the economy and society and the regional demand for achieving eco-friendly growth [34]. Consequently, the supply pressure of airport green development considered from two sources: the pressure on the supply capacity due to economic and social development and the pressure on meeting the demand for green development in the region. These supply pressures have led to changes in the supply state, encompassing the environmental carrying capacity of airports and their operational capabilities. Addressing the deficiencies in the current supply status of green airport development, airport authorities and government departments implement measures such as adjusting human resources and increasing funding inputs to alleviate the supply pressure and improve the supply state. Through these coordinated efforts, a harmonization of supply pressure, supply state, and response is gradually achieved, leading to the effective supply of the demand for green airport development. The specific realization path is shown as Fig 2:

Using the PSR model to construct the supply index system for airport green development, which is shown in Table 2.

**Table 2:** Supply index system for airports green development.

System	Subsystem	Sequence	Indicators	Unit	Attributes
Supply subsystem	P	*y*_1_	Per capita GDP	yuan	Positive
		*y*_2_	The population density	person/km^2^	Positive
		*y*_3_	The reduction rate of energy consumption per unit of GDP	%	Positive
		*y*_4_	The pollutant emission intensity of 10000 yuan of the main revenue	cubic meter /10000 yuan	Negative
	S	*y*_5_	The reduction rate of energy consumption per passenger	%	Positive
		*y*_6_	The proportion of airport public transportation facility guarantee personnel	%	Positive
		*y*_7_	Airport Service Evaluation Score	points	Positive
		*y*_8_	Clearance rate improvement	%	Positive
		*y*_9_	Runway capacity	sorties	Positive
		*y*_10_	Airport per capita income	10000 yuan	Positive
	R	*y*_11_	Green coverage rate	%	Positive
		*y*_12_	The proportion of pollution control investment in GDP	%	Positive
		*y*_13_	The proportion of urban aviation transportation industry investment to GDP	%	Positive

### 3.3. Synergistic evolutionary model of the SDCS

In the field of green development technology, synergistic evolution plays a crucial role in analyzing the interplay among green development elements and investigating their combined effects. Zhang and colleagues developed a composite indicator system for the high-quality development of the ecosystem-industry interface to address the conflict between industrial progress and environmental conservation. They analyzed the interactions among indicators using the Lotka-Volterra model and established a complex network model to depict indicator relationships as a complex system for social network analysis [35]. Yu develops a quantum evolutionary game model rooted in the evolutionary stabilization strategies of key players in the rural new energy industry to investigate the entanglement mechanism among village collectives in the new energy enterprise domain [36]. In order to promote the transformation and development of green energy in rural areas, they further constructed a tripartite evolutionary game model of the government, new energy enterprises and farmers, and analyzed the evolutionary paths of the tripartite players in the process of energy industry transformation [37].

In Synergetics, the order parameters are the crucial factors that drive the orderly development of a system. Following the principle of the role of ordinal covariates in the system, Haken employs mathematical techniques to treat these system parameters. He constructs evolution equations to analyze the synergistic evolution process of composite systems and proposes the adiabatic elimination method to identify the order parameters of composite systems.

Denoting the two subsystems of the composite system by *q*_1_ and *q*_2_, and assuming that *q*_1_ is the order parameter of the system, the interaction and evolutionary relationship between the two subsystems can be expressed by the equation of motion as follows:

$$\frac{dq_{1}}{dt}=-\gamma_{1}q_{1}-aq_{1}q_{2}$$ (1)

$$\frac{dq_{2}}{dt}=-\gamma_{2}q_{2}+b{q_{1}}^{2}$$ (2)

Since the Haken model involves physics-related settings, it primarily operates with continuous-type stochastic variables. However, when applying Eqs (1) and (2) to the analysis of the composite system of supply and demand, they need to be discretized:

$$q_{1}(t)=(1-\gamma_{1})q_{1}(t-1)-aq_{1}(t-1)q_{2}(t-1)$$ (3)

$$q_{2}(t)=(1-\gamma_{2})q_{2}(t-1)+bq_{1}{\left(t-1\right)}^{2}$$ (4)

In the formula, the parameters *a* and *b* represent the control parameters of the interaction strength between subsystems *q*_1_ and *q*_2_. When *a* > 0, there is no positive synergistic effect between *q*_1_ and *q*_2_, and *q*_2_ impedes the evolution process of *q*_1_. When *a* < 0, there is a positive synergistic effect between *q*_1_ and *q*_2_, and *q*_2_ promotes the evolution of *q*_1_ to a higher level. When *b* > 0, *q*_1_ promotes the evolution of *q*_2_. When *b* < 0, *q*_1_ impedes *q*_2_, which is detrimental to the creation of synergistic effects. *γ*_1_ and *γ*_2_ denote the damping coefficients of subsystems *q*_1_ and *q*_2_ respectively. When *γ*_1_ < 1, subsystem *q*_1_ forms a stable and orderly dissipative structure, which is conducive to the further evolution of the system. On the contrary, if *γ*_1_ ≥ 1, the structure of subsystem *q*_1_ is in a disordered and chaotic state, making it difficult to produce synergistic effects. Similarly, when *γ*_2_ < 1, the dissipative structure established by subsystem *q*_2_ can promote the synergistic evolution of the subsystems. On the contrary, if *γ*_2_ ≥ 1, the evolution of subsystem *q*_2_ is adversely affected by the disorderly structure.

Based on the results of the orderliness measurement of the supply-demand composite system, the parameters of the motion equation are solved to determine whether the "adiabatic approximation assumption" is satisfied. If the conditions *γ*_2_ > 0 and *γ*_2_ ≫ |*γ*_1_| are met, the composite system satisfies the adiabatic approximation condition. In this scenario, subsystem *q*_1_ becomes the order parameter driving the synergistic evolution of the composite system.

When the Eqs (1) and (2) satisfy the adiabatic approximation assumption, it implies that the subsystem *q*_1_ dominates the synergistic evolution of the SDCS. At this point, the influence of *q*_2_ becomes negligible, let *dq*_2_(*t*) / *dt* = 0, the order parameter *q*_1_ remains unchanged, and by combining the motion equation, the evolution equation of the system’s order parameter can be determined:

$$\frac{dq_{1}(t)}{dt}=-\gamma_{1}q_{1}(t)-\frac{ab}{\gamma_{2}}q_{1}{\left(t\right)}^{3}$$ (5)

Further integrating *dq*_1_(*t*) / *dt* by the negative sign leads to the potential function of the system, which effectively determines the development trend of the system:

$$\nu =\frac{1}{2}\gamma_{1}q_{1}{\left(t\right)}^{2}+\frac{ab}{4\gamma_{2}}q_{1}{\left(t\right)}^{4}$$ (6)

In Eq (5), by setting *dq*_1_(*t*) / *d*_*t*_ = 0 and combining it with Eq (6), we can solve for the stabilization point [*q*_1_*(*t*), *ν*(*q*_1_*(*t*))] of the synergistic development of the supply-demand composite system. When the product of *a*, *b*, *γ*_1_ and *γ*_2_ is positive, the evolution equation has and only has a unique solution (*q*_1_*(*t*) = 0) corresponding to a stable point on the potential function. However, when the product of *a*, *b*, *γ*_1_ and *γ*_2_ is negative, there are three solutions (*q*_1_*(*t*) = 0, ${q_{1}}^{*}(t)=\sqrt{\left|\frac{\gamma_{1}\gamma_{2}}{ab}\right|}$ and ${q_{1}}^{*}(t)=-\sqrt{\left|\frac{\gamma_{1}\gamma_{2}}{ab}\right|}$) to the evolution equation, corresponding to three stable points on the potential function. When the point on the potential function is in a non-equilibrium position, it has a tendency to return to the steady state. Additionally, the state of the point is also affected by the distance between it and the steady state. From this, the state evaluation function of the system can be defined:

$$d=\sqrt{{\left[q_{1}(t)-{q_{1}}^{*}(t)\right]}^{2}+{\left[\nu (q_{1}(t))-\nu ({q_{1}}^{*}(t))\right]}^{2}}$$ (7)

The larger *d* is, the farther the current state is from the steady state, which represents a lower level of synergistic development between the supply and demand sides of the airport’s green development. On the contrary, the smaller *d* is, the higher the level of synergistic development between the supply and demand sides of the airport’s green development [38].

## Experimental results, analysis and discussion

### 4.1. Study area and its institutional background

CAN stands as one of China’s three prominent gateway complex hub airports. However, its rapid development in recent years has resulted in a considerable increase in air traffic, leading to various economic, social, and environmental challenges. In this context, CAN actively aligns with the strategic planning for green airport construction. Over the next five years, CAN will concentrate on enhancing the green operation management system, implementing low-carbon and high-efficiency operation requirements, among other initiatives, to advance green airport construction.

Examining the mutual interaction and coordinated evolutionary trends between the supply and demand aspects of green development in CAN aids relevant departments in comprehending the current state of airport green development. This insight enables the implementation of targeted measures to establish a high-level coordinated pattern for the supply and demand of airport green development. Accordingly, this paper uses CAN as a case study to investigate the synergistic evolution process of supply and demand in the context of airport green development.

### 4.2. Data preprocessing

The empirical analysis involved collecting indicator data of CAN spanning from 2008 to 2019. The statistical data sources are from the annual report of the CAN, the airport service evaluation report issued by the Civil Aviation Passenger Service Evaluation, the Civil Aviation Administration website, and the flight operation efficiency report. Part of the data comes from existing research on indicator systems for green development of CAN [39, 40].

#### 4.2.1. The orderliness of the DSS and SSS

The orderliness of a system reflects the organic connection between its elements and the hierarchical structure it exhibits. This paper employs the entropy weighting method to determine the objective weights of indicators. Subsequently, the degree of orderliness of the DSS and SSS is measured using the linear weighting method.

Measuring the DSS orderliness *U*_*X*_(*t*) as an example, the measurement of the SSS orderliness *U*_*Y*_(*t*) follows a similar process. The specific steps for both measurements are as follows [41]: Step1. Constructing the entropy evaluation matrix: *X* = (*X*^1^, *X*^2^, …, *X*^*n*^), *X*^*j*^ = (*x*_1*j*_, *x*_2*j*_, …, *x*_*mj*_)^*T*^ ∀*j* ∈ [*1*, *n*]. In the formula provided, *x*_*tj*_ ∀*t* ∈ [*1*, *m*] is for the indicator *j* in year *t*. Step2. The indicator data were standardized using the efficacy function. Standardized data are obtained using the formula $\frac{x_{tj}-{\text{min}}_{t}(x_{tj})}{{\text{max}}_{t}(x_{tj})-{\text{min}}_{t}(x_{tj})}$ for benefit-based indicators and the formula $\frac{{\text{max}}_{t}(x_{tj})-x_{tj}}{{\text{max}}_{t}(x_{tj})-{\text{min}}_{t}(x_{tj})}$ for cost-based indicators. Step3. calculate the entropy weight *ω*_*j*_ for the indicator $j:\omega_{j}=\frac{1-H_{j}}{\sum_{j=1}^{n}\left(1-H_{j}\right)}$. In the formula provided, $H_{j}=-k\sum_{t=1}^{m}f_{tj}\cdot \text{ln}f_{tj}$, $f_{tj}=r_{tj}/\sum_{t=1}^{m}r_{tj}$ and *k* = 1 / ln *m*. Step4. Calculate the degree of ordering of the DSS using linear weighting:$U_{X}(t)=\sum_{j=1}^{m}\omega_{j}\times r_{tj}\;(t=1,2,\ldots ,m)$.

#### 4.2.2. Screening of supply-demand synergy indicators

Synergy is a crucial concept that encompasses the interrelationship between systems. To study this interrelationship, this paper collected a substantial amount of indicator data. However, it is inevitable that these indicators may overlap, leading to potential deviations in the results. To address these challenges, it is necessary to screen and select synergistic indicators of airport green development supply and demand.

The calculation method for the synergy degree of the SDCS is defined by considering the static coordination level, historical evolution direction, and magnitude of orderliness for each subsystem: $SD=\sum_{t=2}^{m}(\alpha \sqrt{U_{X}(t)\cdot U_{Y}(t)}+\beta \sqrt{\theta \frac{\nabla U_{X}(t)}{U_{X}(t-1)}\cdot \frac{\nabla U_{Y}(t)}{U_{Y}(t-1)}})$ [42]. Where *α*, *β* represent the weights of the subsystem ordering degree and the rate of change of the subsystem ordering degree in affecting the overall level of synergistic development of the system respectively, and each of them is taken as 0.5 in the absence of special circumstances. *θ* is the discriminant coefficient. When two subsystems develop in opposite directions or one of them is stagnant, the product of the rates of change is less than or equal to 0, the synergistic effect is weakened, and *θ* is 0. On the contrary, when two subsystems develop in the same direction, the product of the rates of change is greater than 0, the synergistic effect is enhanced, and *θ* is 1.

To deeply explore the interaction relationship between the SSS and DSS and analyze their synergistic evolution trend, this paper aims to maximize the synergy degree of the SDCS, and considers the number of indicators and adiabatic approximation conditions as constraints. Based on these considerations, a screening and optimization model of the synergistic indicators of airport green development is constructed:

maxSD

$$s.t.\left\{\begin{array}{c} n_{x}^{\text{min}}\leq \sum_{j=1}^{n_{x}}x_{j}\leq n_{x}^{\text{max}}\\ n_{y}^{\text{min}}\leq \sum_{j=1}^{n_{y}}y_{j}\leq n_{y}^{\text{max}}\\ \gamma_{2}>0\\ \gamma_{2}>>|\gamma_{1}| \end{array}\right.$$

In the formula, *x*_*j*_ and *y*_*j*_ represent the 0–1 variables of the screening results of each indicator in the demand index system and the supply index system, respectively. $n_{x}^{\text{min}}$ and $n_{x}^{\text{max}}$ represent the lower limit and upper limit of the number of synergistic indicators of airport green development demand, respectively. Similarly,$n_{y}^{\text{min}}$ and $n_{y}^{\text{max}}$ represent the lower and upper limit of the number of synergistic indicators of airport green development supply, respectively. Furthermore, *γ*_1_ and *γ*_2_ are the parameters of the motion equation. It is important to note that when the adiabatic approximation constraint is satisfied, *γ*_2_ is positive and significantly larger than |*γ*_1_|, typically by at least one order of magnitude.

### 4.3. Optimization results of supply-demand synergy indicators screening

Given the extensive number of indicators in the index system, the feasible solution space becomes substantial during indicator selection. The problem is highly intricate, making it challenging to attain the optimal solution by exploring all feasible solutions within polynomial time. To address this challenge, this paper designs a genetic algorithm with an elite retention strategy to solve the problem [43]. Additionally, the individual with the optimal fitness is selected after each genetic operation to perform the simulated annealing operation. This enhances the algorithm’s ability to globally search for near-optimal solutions that meet the constraints within a limited number of iterations, and helps in reducing the probability of the algorithm getting stuck in local optimal solutions [44]. In the course of this iterative process, the supply-demand synergy indicators are identified.

**Fig 3:**
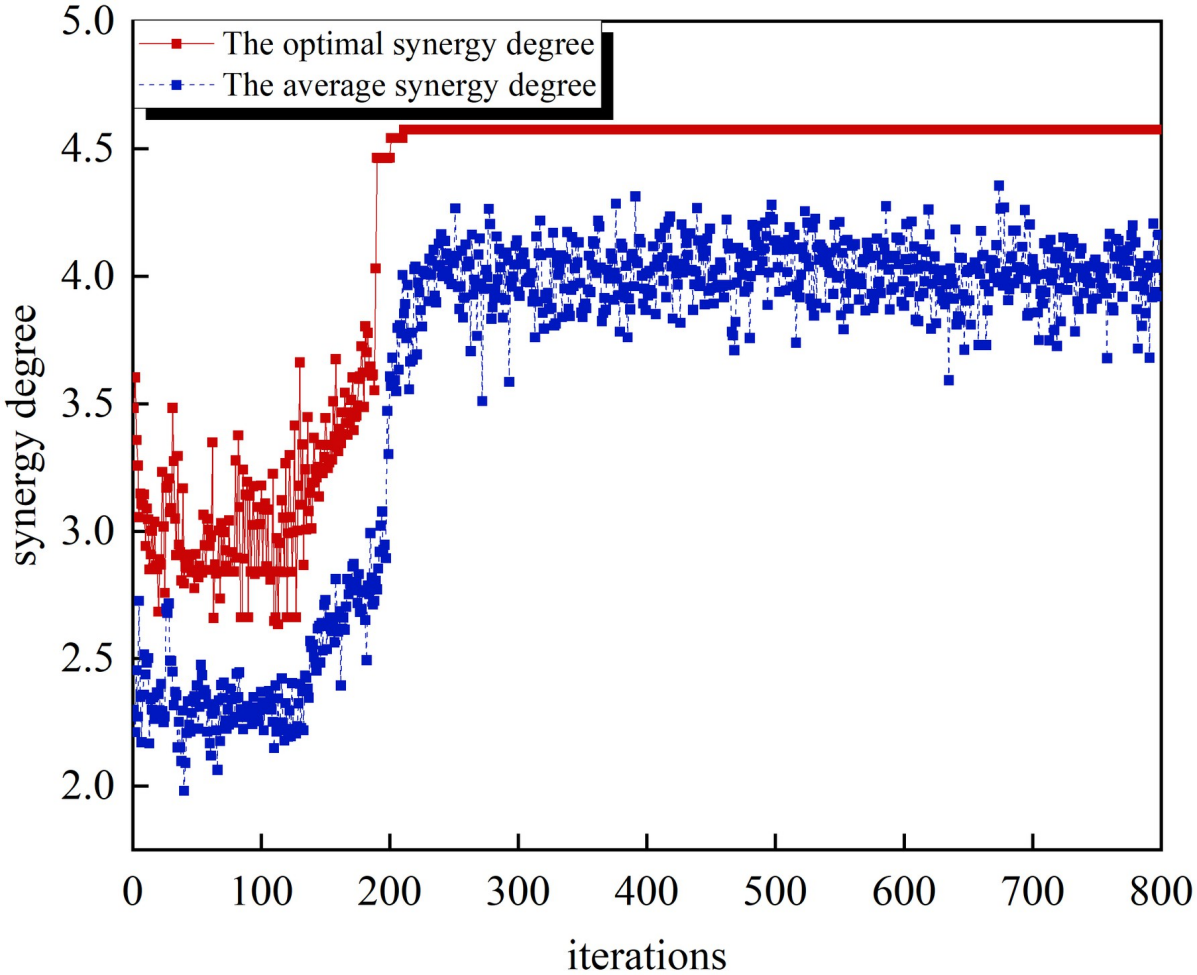
The process diagram of simulated annealing genetic algorithm optimization.

To ensure that the screened supply and demand indicators effectively reflect the criterion attributes for the airport green development, and to prevent homogenization of the indicator screening results, this paper set the values of $n_{x}^{\text{min}}$ and $n_{y}^{\text{min}}$ to 5, while $n_{x}^{\text{max}}$ and $n_{y}^{\text{max}}$ are defined as the total number of indicators in the respective supply and demand index systems. The simulated annealing genetic algorithm was used to solve the optimization model for screening supply-demand synergy indicators, setting the population size at 100, the number of iterations of the genetic algorithm to 800, the crossover probability at 0.9, the variance probability at 0.1, the elite retention rate 0.1, the initial temperature of simulated annealing 1000°C, the temperature attenuation coefficient 0.95, and the number of cycles 100. The optimization process is shown as Fig 3:

Based on Fig 3, the algorithm exhibits convergence after approximately 200 generations following the preliminary exploration of optimization seeking. The optimal synergy degree achieved through this process is 4.575. The corresponding supply-demand synergy indicators and their weights are iterated and shown in Table 3:

**Table 3:** The synergistic indicators of supply and demand for green development in CAN.

System	Subsystem	Indicators	Weight
DSS	R	*x*_1_	9.66%
		*x*_3_	20.26%
		*x*_4_	7.61%
	E	*x*_5_	7.62%
		*x*_7_	9.30%
		*x*_8_	10.88%
	O	*x*_13_	10.31%
		*x*_14_	13.23%
		*x*_15_	11.13%
SSS	P	*y*_1_	22.97%
		*y*_2_	15.34%
	S	*y*_7_	20.46%
		*y*_9_	15.40%
	R	*y*_11_	13.60%
		*y*_13_	12.23%

Based on the entropy weighting results of the synergistic indicators of supply and demand, the orderliness of the CAN’s SDCS is evaluated. The measurement result is displayed in Table 4.

**Table 4:** The orderliness of the CAN’s SSS and DSS.

SSS	Year	2008	2009	2010	2011	2012	2013
	Orderliness	0.5899	0.6194	0.5744	0.5608	0.5690	0.5008
	Year	2014	2015	2016	2017	2018	2019
	Orderliness	0.4588	0.3951	0.4641	0.4374	0.4809	0.5268
DSS	Year	2008	2009	2010	2011	2012	2013
	Orderliness	0.0435	0.1190	0.3249	0.4029	0.4649	0.5137
	Year	2014	2015	2016	2017	2018	2019
	Orderliness	0.5220	0.5735	0.7019	0.7733	0.8446	0.9336

### 4.4. Co-evolutionary results of the CAN’s SDCS

This paper aims to elucidate the central role of the SSS and DSS in the green development of airports and assess their respective contributions to achieving coordinated and environmentally friendly transformation of airports. The study focuses on the period from 2008 to 2019, with the end of the Twelfth Five-Year Plan serving as a significant time node. The Haken model is utilized to investigate the synergistic evolution mechanism of the CAN’s SDCS in two distinct phases, namely 2008–2015 and 2016–2019.

#### 4.4.1. The Haken model results for the first stage (2008–2015)

Utilizing the outcomes derived from the evaluation of the orderliness of the SSS and DSS during the period 2008 to 2015, the evolution of the synergistic development of the two subsystems was analyzed by using Eqs (3) and (4) with the DSS and the SSS as the order parameter, respectively. The equations of motion were solved using Eviews13.0, and the results are shown in Table 5.

**Table 5:** The Haken model results for the first stage.

Assumptions	Equation of motion	Conclusions
$①\left\{\begin{array}{c} q_{1}=U_{X}\\ q_{2}=U_{Y} \end{array}\right.$	$\begin{array}{l} U_{X}(t)=1.02044U_{X}(t-1)-0.214786U_{X}(t-1)U_{Y}(t-1)\\ \;\;\;\;\;\;\;\;\;\;\;\;\;\;(22.1343^{***})\;\;\;\;\;\;\;\;\;\;\;\;\;\;\;\;\;\;(1.693373^{*})\\ \;\;\;\;\;\;\;\;\;\;\;\;\;\;\;\;\;R^{2}=0.8474\;\;\;\;\;\;\;\;\;\;\;\;\;\;\;\;\;\;\;\;\;Adjusted\;R^{2}=0.8169\\ U_{Y}(t)=0.870392U_{Y}(t-1)+0.389308U_{X}{\left(t-1\right)}^{2}\\ \;\;\;\;\;\;\;\;\;\;\;\;\;\;\;\;\;(11.35673^{***})\;\;\;\;\;\;\;\;\;\;\;\;\;\;\;(4.138483^{***})\\ \;\;\;\;\;\;\;\;\;\;\;\;\;\;\;\;\;R^{2}=0.9276\;\;\;\;\;\;\;\;\;\;\;\;\;\;\;\;\;\;\;\;\;Adjusted\;R^{2}=0.9131\\ \gamma_{1}=-0.02044;\;\gamma_{2}=0.129608;\;a=0.214786;\;b=0.389308 \end{array}$	*a*. The motion equation is valid *b*. Satisfy the adiabatic approximation assumption *c*. Model assumptions are valid, where *U*_*X*_ is the order parameter
$②\left\{\begin{array}{c} q_{1}=U_{Y}\\ q_{2}=U_{X} \end{array}\right.$	$\begin{array}{l} U_{Y}(t)=0.219974U_{Y}(t-1)+1.73597U_{Y}(t-1)U_{X}(t-1)\\ \;\;\;\;\;\;\;\;\;\;\;\;\;\;\;\;\;\;(0.22989)\;\;\;\;\;\;\;\;\;\;\;\;\;\;\;\;\;\;\;\;\;\;\;\;\;(-0.95216)\\ \;\;\;\;\;\;\;\;\;\;\;\;\;\;\;\;\;\;R^{2}=0.7288\;\;\;\;\;\;\;\;\;\;\;\;\;\;\;\;\;\;\;\;Adjusted\;R^{2}=0.6745\\ U_{X}(t)=1.00153U_{X}(t-1)-0.19573U_{Y}{\left(t-1\right)}^{2}\\ \;\;\;\;\;\;\;\;\;\;\;\;\;\;\;\;\;\;(29.0757^{***})\;\;\;\;\;\;\;\;\;\;\;\;\;\;\;\;\;\;(-1.84697^{*})\\ \;\;\;\;\;\;\;\;\;\;\;\;\;\;\;\;\;\;R^{2}=0.8573\;\;\;\;\;\;\;\;\;\;\;\;\;\;\;\;\;\;\;\;\;Adjusted\;R^{2}=0.8287\\ \gamma_{1}=0.78003;\;\gamma_{2}=-0.00153;\;a=-1.73597;\;b=-0.19573\; \end{array}$	*a*. The equation of motion is not valid *b*. Not satisfying the adiabatic approximation assumption *c*. The model assumptions are not valid

Note: () represents the t-test value, *, **, ***respectively indicate significant levels of 0.1, 0.05, and 0.01, none * indicates insignificant

As indicated in Table 5, the motion equation ① is well fitted, and the t-tests conducted on the four estimated parameters yielded significant results, further affirming the establishment of the motion equation. Furthermore, it is noteworthy that *γ*_2_ ≫ |*γ*_1_| satisfies the adiabatic approximation assumption and validates the modeling assumption. The motion equation ② fitted generally. However, it is important to note that the t-tests conducted on the estimated parameters, specifically *γ*_1_ and *a*, do not yield significant results, rendering the motion equation invalid. Moreover, the condition *γ*_2_ < |*γ*_1_| implies a violation of the adiabatic approximation assumption, thereby invalidating the underlying model assumption.

According to the results of solving the Haken model, the model assumption ① is valid, based on the parameters of the solved motion equations, which can be further calculated using Eqs (5) and (6):

the system evolution equation:

$$\frac{dU_{X}(t)}{dt}=0.02044U_{X}(t)-0.64516U_{X}{\left(t\right)}^{3}$$

and the system potential function:

$$\nu =-0.01022U_{X}{\left(t\right)}^{2}+0.16129U_{X}{\left(t\right)}^{4}$$

Setting *dU*_*X*_(*t*) / *dt* = 0 allows for the determination of the potential function for the three solutions: *U*_X_*(*t*) = 0, *U*_X_*(*t*) = 0.1780, and *U*_X_*(*t*) = −0.1780. As the SSS and DSS are objective, the system’s evolution equation exclusively accounts for *U*_*X*_(*t*) > 0. Based on the potential function for these three solutions, the stability point of the system is determined to be (0.1780, -0.00016). Subsequently, by applying Eq (7), the evaluation function’s value for any specific state of the SDCS can be calculated as follows:

$$d=\sqrt{{\left[U_{X}(t)-0.1780\right]}^{2}+{\left[\nu (U_{X}(t))+0.00016\right]}^{2}}$$

#### 4.4.2. The Haken model results for the second stage (2016–2019)

Based on the outcomes derived from the evaluation of the orderliness of the SSS and DSS during the period 2016 to 2019, the evolution results of the synergistic development of the two subsystems are shown in Table 6.

**Table 6:** The Haken model results for the second stage.

Assumptions	Equation of motion	Conclusions
$①\left\{\begin{array}{c} q_{1}=U_{X}\\ q_{2}=U_{Y} \end{array}\right.$	$\begin{array}{l} U_{X}(t)=0.222303U_{X}(t-1)+1.06153U_{X}(t-1)U_{Y}(t-1)\\ \;\;\;\;\;\;\;\;\;\;\;\;\;\;\;\;\;\;(0.461726)\;\;\;\;\;\;\;\;\;\;\;\;\;\;\;\;\;\;\;\;\;\;\;\;(-1.7138)\\ \;\;\;\;\;\;\;\;\;\;\;\;\;\;\;\;\;\;R^{2}=0.7824\;\;\;\;\;\;\;\;\;\;\;\;\;\;\;\;\;\;\;\;\;Adjusted\;R^{2}=0.5648\\ U_{Y}(t)=-0.057U_{Y}(t-1)+0.157001U_{X}{\left(t-1\right)}^{2}\\ \;\;\;\;\;\;\;\;\;\;\;\;\;\;\;\;\;\;(20.00767^{***})\;\;\;\;\;\;\;\;\;\;\;\;\;\;\;\;\;(0.817459)\\ \;\;\;\;\;\;\;\;\;\;\;\;\;\;\;\;\;\;R^{2}=0.9973\;\;\;\;\;\;\;\;\;\;\;\;\;\;\;\;\;\;\;\;\;Adjusted\;R^{2}=0.9945\\ \gamma_{1}=0.777697;\;\gamma_{2}=-0.057;\;a=-1.06153;\;b=0.157001 \end{array}$	*a*. The equation of motion is not valid *b*. Not satisfying the adiabatic approximation assumption *c*. The model assumptions are not valid
$②\left\{\begin{array}{c} q_{1}=U_{Y}\\ q_{2}=U_{X} \end{array}\right.$	$\begin{array}{l} U_{Y}(t)=0.958458U_{Y}(t-1)+0.30651U_{Y}(t-1)U_{X}(t-1)\\ \;\;\;\;\;\;\;\;\;\;\;\;\;\;\;\;\;\;(66.35266^{***})\;\;\;\;\;\;\;\;\;\;\;\;\;\;\;\;(-9.80888^{***})\\ \;\;\;\;\;\;\;\;\;\;\;\;\;\;\;\;\;\;R^{2}=0.9999\;\;\;\;\;\;\;\;\;\;\;\;\;\;\;\;\;\;\;\;\;Adjusted\;R^{2}=0.9999\\ U_{X}(t)=0.536825U_{X}(t-1)+0.389114U_{Y}{\left(t-1\right)}^{2}\\ \;\;\;\;\;\;\;\;\;\;\;\;\;\;\;\;\;\;(3.560695^{***})\;\;\;\;\;\;\;\;\;\;\;\;\;\;\;\;(3.402794^{***})\\ \;\;\;\;\;\;\;\;\;\;\;\;\;\;\;\;\;\;R^{2}=0.9319\;\;\;\;\;\;\;\;\;\;\;\;\;\;\;\;\;\;\;\;\;Adjusted\;R^{2}=0.8638\\ \gamma_{1}=0.041542;\;\gamma_{2}=0.463175;\;a=-0.30651;\;b=0.389114\; \end{array}$	*a*. The motion equation is valid *b*. Satisfy the adiabatic approximation assumption *c*. Model assumptions are valid, where *U*_*Y*_ is the order parameter

Note: () represents the t-test value, *, **, ***respectively indicate significant levels of 0.1, 0.05, and 0.01, none * indicates insignificant

As indicated in Table 6, the degree of fit of the DSS orderliness in motion equation ① is poor. And the t-tests conducted on the estimated parameters, specifically *γ*_1_, *a* and *b*, do not yield significant results, rendering the motion equation invalid. Furthermore, the condition *γ*_2_ < 0 implies a violation of the adiabatic approximation assumption, thereby invalidating the underlying model assumptions. The motion equation ② is well fitted, and the t-tests conducted on all four estimated parameters have yielded significant results, thereby validating the motion equation. In this context, it is noteworthy that *γ*_2_ ≫ |*γ*_1_|, satisfying the adiabatic approximation assumption and confirming the validity of the model assumption.

According to the results of solving the Haken model, the model assumption ② is valid, based on the parameters of the solved equations of motion, which can be further calculated using Eqs (5) and (6):

the system evolution equation:

$$\frac{dU_{Y}(t)}{dt}=-0.041542U_{Y}(t)+0.2575U_{Y}{\left(t\right)}^{3}$$

and the system potential function:

$$\nu =0.020771U_{Y}{\left(t\right)}^{2}-0.06437U_{Y}{\left(t\right)}^{4}$$

Setting *dU*_*Y*_(*t*) / *dt* = 0 allows for the determination of the potential function for the three solutions:*U*_Y_*(*t*) = 0, *U*_Y_*(*t*) = 0.40166 and *U*_Y_*(*t*) = −0.40166, and the stability point of the system is further determined as(0.40166, 0.00168). Subsequently, by applying Eq (7), the evaluation function’s value for any specific state of the SDCS can be calculated as follows:

$$d=\sqrt{{\left[U_{Y}(t)-0.40166\right]}^{2}+{\left[\nu (U_{Y}(t))-0.00168\right]}^{2}}$$

In order to capture the evolving dynamics of the CAN’s SDCS, the *d* value is computed based on the state evaluation function for the two distinct phases, and then subjected to normalization using the extremum method. To address the boundary issue involving values of 0 and 1, a precautionary approach is taken where the original evaluation value’s maximum is increased by 10%, and the minimum is decreased by 10% prior to the normalization process. The results are illustrated in Table 7.

**Table 7:** The synergistic evaluation values and scores for the CAN’s SDCS.

Year	2008	2009	2010	2011	2012	2013
Evaluation value	0.4122	0.4418	0.3967	0.3830	0.3913	0.3228
Synergistic score	0.4454	0.3697	0.4850	0.5201	0.4988	0.6741
Year	2014	2015	2016	2017	2018	2019
Evaluation value	0.2809	0.2171	0.3003	0.3718	0.4433	0.5330
Synergistic score	0.7813	0.9445	0.7316	0.5487	0.3658	0.1363

### 4.5. The two-stage synergistic evolutionary analysis of the CAN’s SDCS

The identification results of the order parameter indicate that the order parameter that dominate the synergistic evolution of the SDCS shift from the DSS in the first stage to the SSS in the second stage. This variation in order parameter signifies that the development of the complex system depends on the nodal support effects produced by the main driving factors over time, rather than the development of a single subsystem. The stable solution of the SDCS has been transformed from *U*_X_*(*t*) = 0.1780 in the first stage to *U*_Y_*(*t*) = 0.40166 in the second stage. This advancement marks a significant milestone, underscoring the entry of the SDCS into a new phase of co-evolution.

Based on the parameter solution outcomes from the two-stage Haken model, it is evident that the parameters in both stages adhere to the conditions *γ*_1_ < 1 and *γ*_2_ < 1. These results indicate that the SSS and the DSS have established a reinforcing positive feedback mechanism. This collaborative feedback process has led to the formation of a stable and organized dissipative structure, fostering an environment conducive to the continued synergistic evolution of the composite system.

The analysis of parameter values of *a* in the first stage and *b* in the second stage offers insights into the influence of the SSS on the DSS. In the first stage, *a* = 0.214786 is positive, which suggests that CAN’s supply structure and capacity of green development impeded the progression of the green development demand to a higher level. In the second stage, *b* = 0.389114 is positive, indicating that CAN’s green development supply in this phase is contributing to the realization of the green development demand. Similarly, the analysis of parameter values of *b* in the first stage and *a* in the second stage offers insights into the influence of the DSS on the SSS. The *b* = 0.389308 is positive in the first stage and the *a* = −0.30651 is negative in the second stage, which indicates that CAN’s green development demand can promote the enhancement of the green development supply capacity within the research interval.

**Fig 4:**
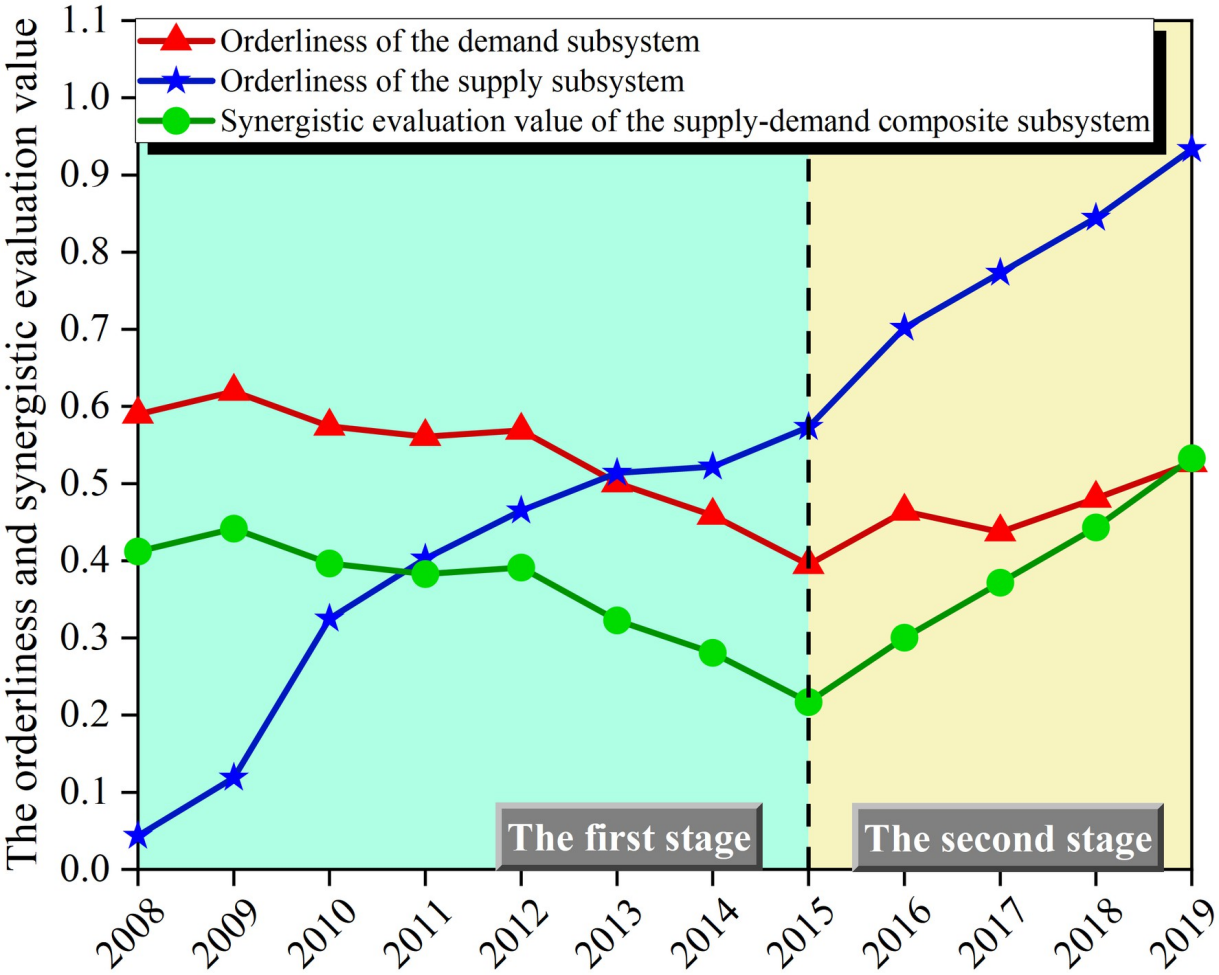
The trends in orderliness and synergistic evaluation values.

Comparing the synergistic evaluation value of the CAN’s SDCS with the orderliness of the SSS and DSS, as shown in Fig 4:

Referring to Fig 4, it is evident that the synergistic evaluation value of the SDCS in the first stage exhibits synchronous changes alongside the orderliness within the DSS. Similarly, the synergistic evaluation value of the SDCS in the second stage exhibits synchronous changes alongside the orderliness within the SSS. Notably, the onset of a new evolutionary stage aligns with the lowest point of the synergistic evaluation value. This observation concurs with the evolutionary pattern depicted by the two-stage order parameters.

**Fig 5:**
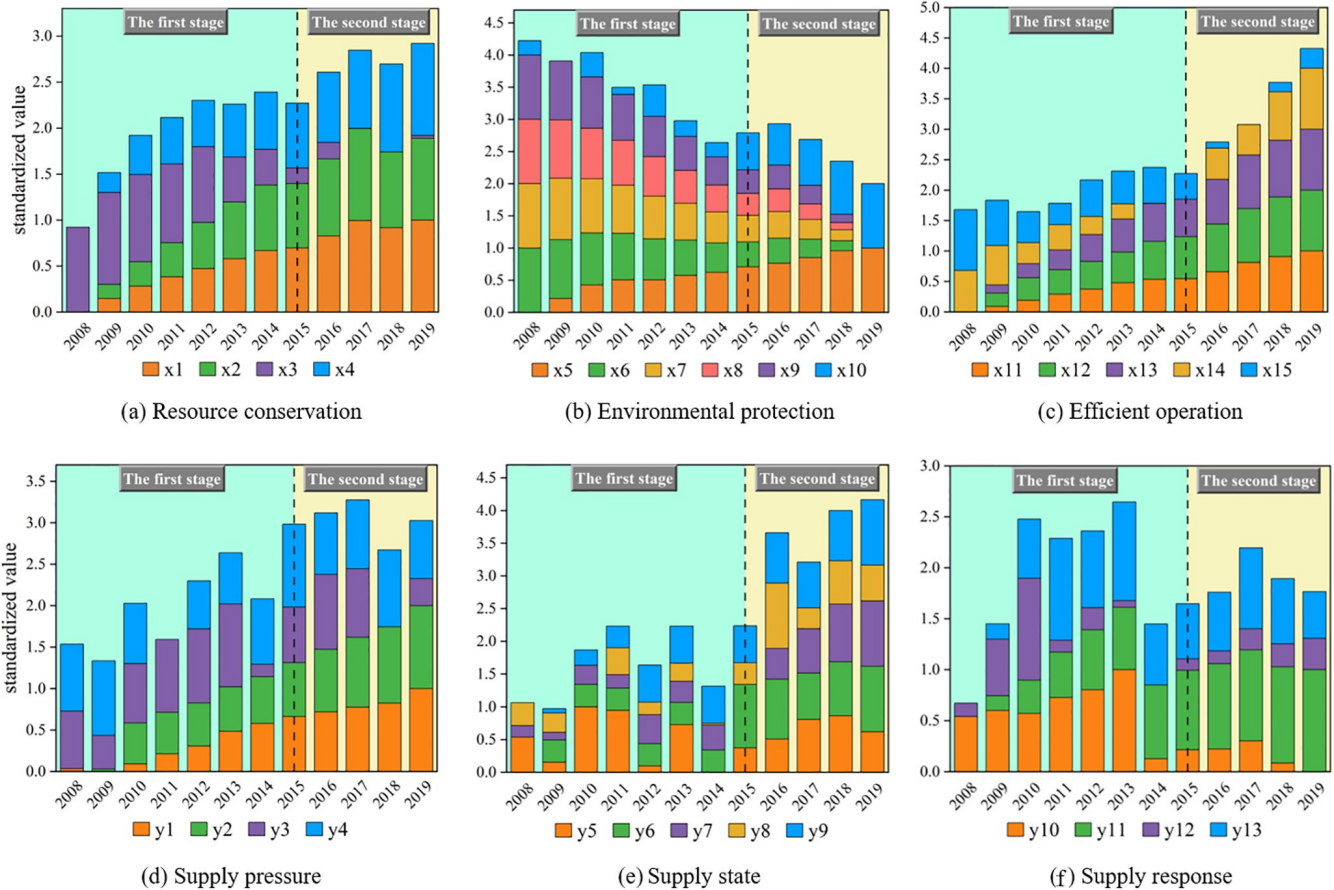
Results of data standardization for supply and demand indicators.

To discern the evolution trend of the orderliness within the SSS and DSS, the data pertaining to CAN’s green development supply and demand indicators within the specified research interval have been standardized, as visually represented in Fig 5:

According to Fig 5, during the study interval, in the macro-environmental context of social development and economic growth, the per capita GDP and urban population density of Guangzhou have been effectively improved, providing a strong guarantee for the continuous increase of the orderliness of the SSS.

Simultaneously, the regional economic radiation, along with the influential role of CAN as a central hub in the region, has propelled a consistent expansion of CAN’s production scale over the years. However, this expansion has introduced certain challenges, including escalated resource consumption, a surge in pollutant gas emission indices, and operational inefficiencies. Consequently, these challenges collectively contributed to a gradual reduction of the orderliness within the DSS between the years 2008 and 2015.

Subsequent to 2015, the development phase of the "13th Five-Year Plan" ushered in a new era of national airport construction. During this period, CAN successfully concluded the second phase of its expansion project, marked by the operational debut of the third runway and Terminal 2. This transformative step has resulted in substantial upgrades and capacity expansions for CAN. This has notably resulted in a reduction in the average taxiing time for aircraft, thereby better aligning with the imperatives of airport green development and significantly contributing to the enhancement of the DSS orderliness.

**Fig 6:**
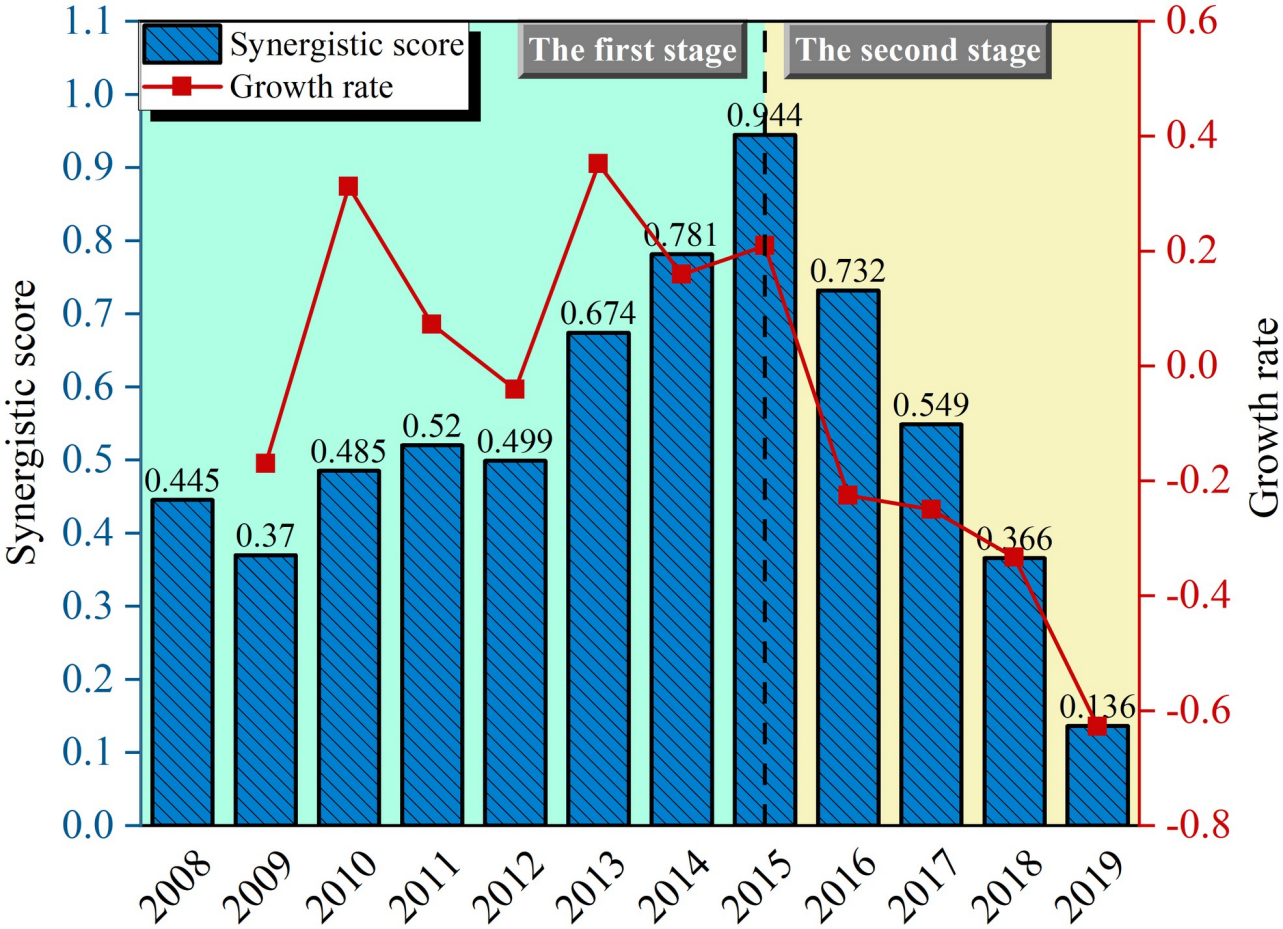
The synergistic score and its growth rate.

The synergistic score and its growth rates are further visualized as shown in Fig 6:

As depicted in Fig 6, the overall synergy level of the CAN’s SDCS in the first stage has been improved, in which, after the fluctuating adjustment in the previous period (2008–2012), the low level of supply capacity for green development and the low-quality demand for green development have already formed a relatively stable low-level ordered structure. Within this structured framework, the smooth and orderly flow of information and resources, represented as energy (including capital, manpower, technology, etc.), ensured the sustained generation of synergistic effects among the subsystems. This cumulative process ultimately culminated in an upward trajectory of composite system synergy. This phase can be classified as a stage of low-level ordered high-level synergy.

In the second stage, the overall synergy level within the SDCS exhibits a gradual decline. This trend signifies a pivotal juncture as China’s development advances into the "13th Five-Year Plan" phase, sparking a process of self-organization and adjustment within the composite system. During this phase, the composite system becomes subject to fresh imperatives arising from the external policy environment, which demands the green transformation of both airport supply and demand for green development. This shift disrupts the preceding low-level synergy that characterized the ordered state of subsystems. The system veers away from its previous steady state due to non-linear effects, ultimately resulting in a decrement in the synergy level. This transition heralds the formation of a new, more advanced, and orderly structure, which belongs to the stage of high-level ordered low-level synergy. The process of adjustment serves as an inevitable trajectory toward achieving heightened synergy level in the future.

### 4.6. Discussion

This paper explores the systematic mechanism and evolutionary process of airport green development from the perspective of supply-demand coordination. In contrast to existing research on airport green development that lacks an integrated analysis of supply-demand relationships, the significance of this study lies in:

1. This study explicates the meaning of the airport green development supply and demand, and proves that the essence of airport green development is fundamentally a coordinated development process involving its supply and demand elements. The evolutionary mechanism adheres to the self-organizing evolutionary characteristics of openness, non-linearity, fluctuations, and departure from equilibrium. This conclusion aligns with Duan’s study on the evolutionary characteristics of ground transportation supply and demand systems [13]. However, the self-organized evolutionary process of distinct systems is influenced by interactions among their internal elements.
2. This study chose indicators for airport green development supply and demand, considering the perspectives of economy, society, environment, and operation. These indicators cover the essential requirements of national development plans for sustainable development, illustrating the crucial role of airport green development in achieving sustainable development goals, aligning closely with the connotations of energy, environment, and economics [45]. In addition, this study analyzes the supply and demand dynamics of airport green development, aiding airport authorities in understanding current supply conditions and identifying gaps in green development. It recommends responsive strategies such as resource conservation, low-carbon emissions, and operational efficiency to ensure effective green development. This approach enhances airport sustainability and drives green innovation, contributing significantly to the green transformation of the civil aviation industry [46].
3. This study distinguishes itself from most related research [23–25] by building an optimized model to screen indicators for the coordination of supply and demand in airport green development. This model is based on the approximation adiabatic conditions of the Haken model. It reveals the interactive relationship and phased coordinated development process of the supply and demand sides in airport green development. The research findings suggest that the optimized indicators effectively characterize various elements of supply and demand. The fitting results of the Haken model are notably good, offering new insights for optimizing supply and demand relationships and coordinating regional green development.

## Conclusions and enlightenment

### 5.1. Conclusions and suggestions

Addressing the limitations of existing studies, which primarily focus on mitigating airport supply-demand contradictions from a unilateral supply or demand standpoint, this paper endeavors to introduce synergistic theory into the examination of the composite system’s synergistic evolution encompassing both supply and demand aspects of airport green development. Drawing on data from the period 2008–2019 pertaining to CAN, the research culminates in the following key findings:

1. This study focuses on airports’ green development policy, and constructs the demand index system for airport green development based on the three dimensions of "Resource-Environment-Operation". Utilizing the PSR model, it establishes the supply index system for airport green development from the three dimensions of "Pressure-State-Response ". On this basis, the entropy weighting method is used to determine the weights of the indicators, and the linear weighting method is used to measure the orderliness of the SSS and DSS. The results effectively characterize the level of supply and demand for airport green development.
2. In order to ensure that the research dataset of this study meets the conditions for the use of the Harken model, a synergy model is integrated to calculate the synergy of the supply-demand composite system. Additionally, an optimization model is established with the objective of maximizing synergy, using the number of indicators and adiabatic approximation conditions as constraints. The model is solved with a simulated annealing genetic algorithm, iteratively extracting the supply and demand synergy indicators for airport green development.
3. According to the development history of CAN, taking the year of the end of the “12th Five-Year Plan” as the time node, a two-stage Haken model is constructed to analyze the synergistic evolution of the SDCS. The outcomes demonstrate a shift in dominance from the DSS, characterized by order parameters, during the initial stage, to the SSS in the subsequent stage. This transition signifies the advancement of the SDCS’s synergistic evolution into a new phase, marked by a transition from a low-level ordered state to a high-level ordered state. However, the attainment of a fully realized high-level synergistic state has not been reached at this juncture.

To further enhance the high-quality collaborative evolution of the supply-demand compound system for airport green development, the following policy suggestions are proposed:

1. Based on the screening and weight allocation results of the CAN green development supply-demand coordination index, the weight ratio of resource conservation and efficient operation indicators in the demand subsystem is relatively large, at 37.53% and 34.67% respectively. Consequently, CAN should concentrate on standardizing the green construction of airport terminals and enhancing the operational management of flights to reduce the resource consumption and enhance the operational efficiency of the airport.
2. The weight ratio of the supply pressure layer and the supply status layer in the supply subsystem is relatively high, at 38.31% and 35.86%, respectively. This indicates the close relationship between the green development supply level of CAN and the airport’s service capability, and its susceptibility to the impact of regional socio-economic development. However, in comparison with the world’s core cities, Guangzhou lacks a prominent advantage in terms of its overall economic scale, economic concentration, and industrial structure hierarchy. Therefore, the local government departments should concentrate on expanding the international service capabilities of airports. They should also enhance the modern transportation system, reinforce the status of being a comprehensive gateway city, and strive towards the goal of establishing a southern international aviation and shipping center.
3. In the research area, there is a significant difference in the growth of orderliness between the supply and demand subsystems, with the development speed of the supply subsystem far exceeding that of the demand subsystem. The relevant department should make reasonable adjustments to the supply structure for the airport green development, enhance investment in airport expansion and construction, and plan the terminals and aprons rationally based on energy conservation, environmental protection, and process optimization. This aims to achieve a short-term increase in the on-time performance of flights and long-term energy conservation and emission reduction.

### 5.2. Enlightenment and prospects

Airports, as crucial air transportation hubs, face various sustainable development challenges due to rapid socio-economic growth and increasing air travel demand. These challenges include resource wastage, emission rises, and operational inefficiencies, intensifying the conflict between supply and demand for airport green development. Exploring the supply-demand relationship in airport green development and finding paths for airport green transformation are urgent and critical topics requiring in-depth investigation. Through analyzing the synergistic evolution process of green development supply and demand sides in CAN, this study gains the following insights and perspectives:

1. Airport greening process is intricately linked to social, economic, environmental, and operational factors, demonstrating a non-linear interaction mechanism among them. This interaction arises from the supply elements ensuring airport green development, encompassing socio-economic benefits, green development principles, and environmental technology levels, and then translated into the airport green development demand to achieve resource conservation, environmental benefits, and operational advantages.
2. The airport green development process involves aligning supply and demand elements to achieve synergistic evolution. The order parameters play a pivotal role in driving the synergistic evolution of the system. Understanding its changes allows us to comprehend the dynamic process of airport green development and provide a reference for future decision-making.
3. Due to the limited nature of data acquisition, this study only focuses on the synergistic evolution research of the supply and demand subsystem of CAN green development. Future research could consider emphasizing the perspective of regional green development, studying the collaborative evolution relationship of supply and demand in airport clusters. This would provide targeted suggestions for coordinating China’s regional green development [47]. Additionally, as digital technology advances, green digital technology will play a crucial role in propelling airport green development. Integrating green and digital technologies can enhance dynamic airport operations management and resource allocation for green initiatives. Future research should prioritize applying green digital technologies to optimize resource utilization, enhance energy efficiency, and mitigate carbon emissions [48].

## Supporting information

S1 Table Data on airport green development supply and demand indicators for Guangzhou Baiyun International Airport, 2008–2019. (DOCX)
