# Supplementary material for: The synergistic evolution of supply-demand composite system for airport green development: A case study in Guangzhou Baiyun International Airport, China
Source: PLoS One. 2024 Apr 30;19(4):e0302303. doi: 10.1371/journal.pone.0302303 (PMC11060580; doi:10.1371/journal.pone.0302303)
Supplement: S1 Table — (DOCX) [file pone.0302303.s001.docx]

Data on airport green development supply and demand indicators for Guangzhou Baiyun International Airport, 2008-2019

1. Data on airport green development demand indicators

1.1 Resource conservation layer indicators

| year | Annual passenger throughput per unit land area (10000 persons/km^2^) | Annual takeoff and landing sorties per unit land area (10000 sorties/km^2^) | Annual average unit passenger comprehensive water consumption (Liter/person) | Annual average unit passenger comprehensive energy consumption (Kilogram standard coal/person) |
| --- | --- | --- | --- | --- |
| 2008 | 285.1126834 | 1.947166667 | 64.73 | 4.498290972 |
| 2009 | 330.991804 | 2.144881944 | 61.27 | 4.266169882 |
| 2010 | 372.8600517 | 2.286208333 | 63.52 | 4.039019905 |
| 2011 | 403.8270293 | 2.425409722 | 67.62 | 3.953852203 |
| 2012 | 431.8372441 | 2.592458333 | 69.14 | 3.952386577 |
| 2013 | 465.2985408 | 2.738909722 | 83.93 | 3.876969644 |
| 2014 | 492.6138059 | 2.862569444 | 88.37 | 3.826355363 |
| 2015 | 502.0008663 | 2.844993056 | 98.18 | 3.735450564 |
| 2016 | 542.2922949 | 3.0224375 | 97.73 | 3.672912464 |
| 2017 | 594.3712239 | 3.231215278 | 105.62 | 3.579946121 |
| 2018 | 570.6077056 | 3.002289308 | 105.66 | 3.46462734 |
| 2019 | 595.6666432 | 3.089616352 | 104.26 | 3.417357811 |

1.2 Environmental protection layer indicators

| year | Annual CO2 emissions per unit passenger throughput (g/person) | Annual CO2 emissions per unit passenger throughput (g/person) | Annual CO emissions (g) | Annual HC emissions (g) | Annual NOx emissions (g) | Annual PM emissions (g) | Noise pollution (dB) |
| --- | --- | --- | --- | --- | --- | --- | --- |
| 2008 | 9.64530924 | 9.64530924 | 1700000 | 175000 | 1970000 | 20500 | 59.1 |
| 2009 | 9.14759139 | 9.14759139 | 1850000 | 183000 | 2130000 | 22200 | 58.1 |
| 2010 | 8.66053269 | 8.66053269 | 2040000 | 202000 | 2350000 | 24500 | 59.8 |
| 2011 | 8.47791471 | 8.47791471 | 2190000 | 218000 | 2510000 | 26200 | 58.6 |
| 2012 | 8.47477209 | 8.47477209 | 2340000 | 233000 | 2660000 | 27900 | 60.3 |
| 2013 | 8.31306187 | 8.31306187 | 2500000 | 249000 | 2840000 | 29800 | 59.2 |
| 2014 | 8.20453389 | 8.20453389 | 2660000 | 265000 | 3000000 | 31600 | 59.1 |
| 2015 | 8.00961433 | 8.00961433 | 2780000 | 277000 | 3140000 | 33000 | 60.7 |
| 2016 | 7.87551911 | 7.87551911 | 2780000 | 277000 | 3120000 | 32900 | 61 |
| 2017 | 7.67617916 | 7.67617916 | 2960000 | 295000 | 3320000 | 34500 | 61.3 |
| 2018 | 7.42891074 | 7.42891074 | 3190000 | 319000 | 3550000 | 37600 | 61.8 |
| 2019 | 7.32755464 | 7.32755464 | 3470000 | 348000 | 3750000 | 40200 | 62.6 |

1.3 Efficient operation layer indicators

| year | Annual passenger throughput (person) | Annual passenger throughput (person) | Annual cargo throughput (ton) | Annual aircraft movements (sorties) | Airport clearance rate (%) | Average airport taxi time (minute) |
| --- | --- | --- | --- | --- | --- | --- |
| 2008 | 33435472 | 33435472 | 685867.9 | 280392 | 82.6 | 15 |
| 2009 | 37048712 | 37048712 | 955269.7 | 308863 | 81.9 | 17.5 |
| 2010 | 40975673 | 40975673 | 1144456 | 329214 | 75.8 | 19.8 |
| 2011 | 45040340 | 45040340 | 1179968 | 349259 | 77.2 | 21.3 |
| 2012 | 48309410 | 48309410 | 1248764 | 373314 | 74.8 | 18.9 |
| 2013 | 52450262 | 52450262 | 1309746 | 394403 | 73.9 | 19.48 |
| 2014 | 54780346 | 54780346 | 1454044 | 412210 | 68.82 | 19 |
| 2015 | 55201915 | 55201915 | 1537759 | 409679 | 68.8 | 20.6 |
| 2016 | 59732147 | 59732147 | 1652215 | 435231 | 79.21 | 23.75 |
| 2017 | 65806977 | 65806977 | 1780423 | 465295 | 78.99 | 24.75 |
| 2018 | 69720403 | 69720403 | 1890560 | 477364 | 84.95 | 23.25 |
| 2019 | 73378475 | 73378475 | 1919927 | 491249 | 89.1 | 21.58 |

2. Data on airport green development supply indicators

2.1 Supply pressure layer indicators

| year | Per capita GDP (10000 yuan) | The population density (person/km^2^) | The reduction rate of energy consumption per unit of GDP (%) | The pollutant emission intensity of 10000 yuan of the main revenue (cubic meter /10000 yuan) |
| --- | --- | --- | --- | --- |
| 2008 | 81941 | 1370 | 4.56 | 2123.957821 |
| 2009 | 79457 | 1390 | 4.01 | 2055.341586 |
| 2010 | 86582 | 1710 | 4.6 | 2185.464844 |
| 2011 | 95830 | 1715 | 4.91 | 2731.908077 |
| 2012 | 103123 | 1727 | 4.94 | 2296.125368 |
| 2013 | 116825 | 1739 | 5.14 | 2267.044828 |
| 2014 | 124088 | 1759 | 3.52 | 2138.077624 |
| 2015 | 130522 | 1816 | 4.52 | 1979.865171 |
| 2016 | 134761 | 1889 | 4.96 | 2173.942592 |
| 2017 | 139246 | 1950 | 4.81 | 2105.756951 |
| 2018 | 142860 | 2005 | 3.24 | 2034.126112 |
| 2019 | 156427 | 2059 | 3.86 | 2204.592104 |

2.2 Supply state layer indicators

| year | Runway capacity (sorties) | Runway capacity (sorties) | The reduction rate of energy consumption per passenger (%) | Airport service evaluation score (points) | Clearance rate improvement (%) | The proportion of airport public transportation facility guarantee personnel (%) |
| --- | --- | --- | --- | --- | --- | --- |
| 2008 | 48 | 48 | 0.019 | 3.7 | 0.005 | 0.786042171 |
| 2009 | 50 | 50 | 0.005 | 3.68 | -0.008 | 0.7965154 |
| 2010 | 55 | 55 | 0.036 | 3.74 | -0.074 | 0.796532507 |
| 2011 | 58 | 58 | 0.034 | 3.71 | 0.018 | 0.796505181 |
| 2012 | 65 | 65 | 0.003 | 3.79 | -0.031 | 0.796509953 |
| 2013 | 65 | 65 | 0.026 | 3.75 | -0.012 | 0.796505797 |
| 2014 | 65 | 65 | -0.0006 | 3.77 | -0.068 | 0.796505478 |
| 2015 | 65 | 65 | 0.013 | 3.64 | -0.0002 | 0.815917682 |
| 2016 | 71 | 71 | 0.018 | 3.8 | 0.151 | 0.814131919 |
| 2017 | 69 | 69 | 0.029 | 3.87 | -0.002 | 0.807770103 |
| 2018 | 71 | 71 | 0.031 | 3.94 | 0.075 | 0.811393604 |
| 2019 | 78 | 78 | 0.022 | 3.98 | 0.049 | 0.816797102 |

2.3 Supply response layer indicators

| year | Airport per capita income (10000 yuan) | Airport per capita income (10000 yuan) | Green coverage rate (%) | The proportion of pollution control investment in GDP (%) | The proportion of urban aviation transportation industry investment to GDP (%) |
| --- | --- | --- | --- | --- | --- |
| 2008 | 202410.4039 | 202410.4039 | 0.0751 | 0.02454114 | 0.00305283 |
| 2009 | 213192.4348 | 213192.4348 | 0.0776 | 0.03837422 | 0.00510564 |
| 2010 | 207809.2146 | 207809.2146 | 0.0807 | 0.05285996 | 0.01096393 |
| 2011 | 236550.6211 | 236550.6211 | 0.0827 | 0.02416514 | 0.01670637 |
| 2012 | 250519.6595 | 250519.6595 | 0.0851 | 0.02736652 | 0.01333452 |
| 2013 | 286619.0645 | 286619.0645 | 0.0855 | 0.02247929 | 0.01627052 |
| 2014 | 126233.9358 | 126233.9358 | 0.0874 | 0.02032536 | 0.01122148 |
| 2015 | 142472.6413 | 142472.6413 | 0.0884 | 0.02393345 | 0.01044222 |
| 2016 | 143784.1936 | 143784.1936 | 0.0893 | 0.02439429 | 0.01094145 |
| 2017 | 158433.2165 | 158433.2165 | 0.0903 | 0.02705638 | 0.01389728 |
| 2018 | 118506.7856 | 118506.7856 | 0.0911 | 0.02766776 | 0.01177866 |
| 2019 | 102988.3843 | 102988.3843 | 0.0921 | 0.03034975 | 0.00929889 |
